# TRIM29 is required for efficient recruitment of 53BP1 in response to DNA double‐strand breaks in vertebrate cells

**DOI:** 10.1002/2211-5463.12954

**Published:** 2020-08-31

**Authors:** Rakkreat Wikiniyadhanee, Tassanee Lerksuthirat, Wasana Stitchantrakul, Sermsiri Chitphuk, Thanyachai Sura, Donniphat Dejsuphong

**Affiliations:** ^1^ Section for Translational Medicine Faculty of Medicine Ramathibodi Hospital Mahidol University Bangkok Thailand; ^2^ Research Center Faculty of Medicine Ramathibodi Hospital Mahidol University Bangkok Thailand; ^3^ Department of Internal Medicine Faculty of Medicine Ramathibodi Hospital Mahidol University Bangkok Thailand

**Keywords:** 53BP1, DT40, etoposide, nonhomologous end joining, TRIM29

## Abstract

Tripartite motif‐containing protein 29 (TRIM29) is involved in DNA double‐strand break (DSB) repair. However, the specific roles of TRIM29 in DNA repair are not clearly understood. To investigate the involvement of TRIM29 in DNA DSB repair, we disrupted *TRIM29* in DT40 cells by gene targeting with homologous recombination (HR). The roles of TRIM29 were investigated by clonogenic survival assays and immunofluorescence analyses. *TRIM29* triallelic knockout (*TRIM29* ^−/−/−/+^) cells were sensitive to etoposide, but resistant to camptothecin. Foci formation assays to assess DNA repair activities showed that the dissociation of etoposide‐induced phosphorylated H2A histone family member X (ɣ‐H2AX) foci was retained in *TRIM29* ^−/−/−/+^ cells, and the formation of etoposide‐induced tumor suppressor p53‐binding protein 1 (53BP1) foci in *TRIM29* ^−/−/−/+^ cells was slower compared with wild‐type (*WT*) cells. Interestingly, the kinetics of camptothecin‐induced RAD51 foci formation of *TRIM29* ^−/−/−/+^ cells was higher than that of *WT* cells. These results indicate that TRIM29 is required for efficient recruitment of 53BP1 to facilitate the nonhomologous end‐joining (NHEJ) pathway and thereby suppress the HR pathway in response to DNA DSBs. TRIM29 regulates the choice of DNA DSB repair pathway by facilitating 53BP1 accumulation to promote NHEJ and may have potential for development into a therapeutic target to sensitize refractory cancers or as biomarker of personalized therapies.

## Graphical abstract

TRIM29 is involved in DNA double‐strand break repair but the specific roles of TRIM29 in DNA repair are not clearly understood. We proposed that TRIM29 regulates the choice of DNA DSB repair pathway by facilitating 53BP1 accumulation to promote NHEJ, possibly through RNF8 or TIP60.

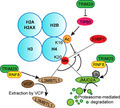

**Abbreviations**
53BP1: tumor suppressor p53‐binding protein 1
alt‐EJ: alternative end joining
ATDC: ataxia‐telangiectasia group D complementing protein
ATM: ataxia‐telangiectasia mutated
BASC: BRCA1‐associated surveillance complex
BRCA1: breast cancer type 1 susceptibility protein
*BRCA1^−/−^*: *BRCA1* knockout
BRCA2: breast cancer type 2 susceptibility protein
*Bsr*: blasticidin S deaminase
cisplatin: cis‐diamminedichloroplatinum
CST: CTC1/STN1/TEN1
CtIP: CtBR‐interacting protein
ddPCR: droplet digital PCR
DDR: DNA damage response
DNA‐PKcs: DNA‐dependent protein kinase catalytic subunit
*DNA*‐*PKcs^−/−/−^*: *DNA‐PKcs* knockout
DSBs: double‐strand breaks
GC: gene conversion
ɣ‐H2AX: phosphorylated H2A histone family member X
H4K16^Ac^: histone H4 acetylated at Lys 16
H4K20^Me2^: histone H4 dimethylated at Lys 20
HR: homologous recombination
*Ku70^−/−^*: *Ku70* knockout
LIG4: DNA ligase 4
*LIG4^−/−^*: *LIG4* knockout
MDC1: mediator of DNA damage checkpoint protein 1
MRN: MRE11/RAD50/NBS1
*Neo*: neomycin phosphotransferase
NHEJ: nonhomologous end joining
PALB2: partner and localizer of BRCA2
*PALB2^−/−^*: *PALB2* knockout
PAXX: paralog of XRCC4 and XLF
PBS: phosphate‐buffered saline
*Puro*: puromycin acetyltransferase
*REV1^−/−^*: *REV1* knockout
RPA: replication protein A
SD: standard deviation
SSA: single‐strand annealing
SSBs: DNA single‐strand breaks
ssDNA: single‐stranded DNA
TIP60: Tat‐interactive protein 60
TOP: topoisomerase
TRIM29: tripartite motif‐containing protein 29
*TRIM29*^−/−/−/+^: *TRIM29* triallelic knockout
*TRIM29*^−/−/+/+^: *TRIM29* biallelic knockout
*TRIM29*^−/+/+/+^: *TRIM29* monoallelic knockout
UV‐C: ultraviolet c
*WT*: wild‐type
XLF: XRCC4‐like factor
XRCC4: X‐ray repair cross‐complementing 4
*XRCC4^−/−^*: *XRCC4* knockout

Radiotherapy and the majority of chemotherapeutic drugs, including topoisomerase (TOP) 1 and 2 inhibitors, induce DNA double‐strand breaks (DSBs) that are considered as one of the most biologically lethal damage to a cell. Unrepaired or mis‐repaired DNA DSBs can cause cell death and genomic instability [1]. In higher eukaryotic cells, homologous recombination (HR) and nonhomologous end joining (NHEJ) are the two predominant pathways, together with alternative end joining (alt‐EJ) and single‐strand annealing (SSA), to repair DNA DSBs [2, 3]. HR precisely repairs DNA DSBs by copying information from a DNA template, which is usually a sister chromatid. At the initial step of HR, the DSB end is resected to generate a 3′ single‐stranded DNA (ssDNA) overhang. The MRE11/RAD50/NBS1 (MRN) complex and CtBR‐interacting protein (CtIP) are also required for this end resection process [4]. Replication protein A (RPA) then coats the 3′ ssDNA overhang to destabilize the DNA secondary structure and to protect 3′ ssDNA overhang from nucleolytic degradation. Subsequently, RAD51 replaces RPA facilitated by breast cancer type 1 susceptibility protein (BRCA1), partner, and localizer of BRCA2 (PALB2) and breast cancer type 2 susceptibility protein (BRCA2) [5, 6]. In contrast to HR, the intact template and intensive resection step are not required for NHEJ. NHEJ is initially triggered by the binding of Ku70/80 heterodimers at DNA DSB sites. Subsequently, other repair proteins, such as DNA‐dependent protein kinase catalytic subunit (DNA‐PKcs), Artemis, X‐ray repair cross‐complementing 4 (XRCC4), XRCC4‐like factor (XLF), newly identified paralog of XRCC4 and XLF (PAXX), and DNA ligase 4 (LIG4), are recruited to DNA DSB sites [7]. HR is relatively active during S and early G2 cell cycle phases, while NHEJ is active throughout all phases [8]. In addition to cell cycle phases, the choice between HR and NHEJ pathways is influenced by types of damaged DNA ends and DNA end resection [9]. For example, intensive DNA end resection required for HR can be suppressed by tumor suppressor p53‐binding protein 1 (53BP1), which is thought to promote NHEJ. 53BP1 not only suppresses HR, but also foster fidelity of HR by limiting mutagenic single‐stranded annealing (SSA), which is triggered by hyper‐resection of DNA ends [10]. However, 53BP1 requires other protein partners to efficiently function in DNA repair because 53BP1 alone is not enough to block DNA end resection or protect DNA ends from nucleolytic activities [11] that draws attention to the identification of novel factors associated with 53BP1‐dependent DNA repair. For instance, researchers have recently discovered a four‐subunit protein complex called shieldin, which is composed of REV7, SHLD1, SHLD2, and SHLD3 [11]. Shieldin complex also promotes many cellular processes that are associated with 53BP1, such as protection of DNA ends, immunoglobulin class switching, and NHEJ [12, 13, 14]. It has been reported that shieldin complex also interacts with CTC1/STN1/TEN1 (CST) complex, which is able to antagonize end resection [15].

It is known that DNA repair partially plays a role in the cancer therapeutic response. Either elevated or reduced activities of DNA repair can determine outcomes of cancer treatments [16]. Upregulation of DNA repair can result in cancer resistance to therapies [9]. Although numerous proteins associated with DNA DSB repair are characterized well, the functions of some identified proteins are not clearly described in DNA DSB repair. Therefore, a better understanding of DNA DSB repair is crucial to identify potential targets to sensitize treatment‐resistant cancers and improve outcomes.

Tripartite motif‐containing protein 29 (TRIM29) is also known as ataxia‐telangiectasia group D complementing protein (ATDC). Structurally, TRIM family proteins contain three conserved domains: a RING‐finger domain, one or two B‐box domains, and a coiled‐coil domain [17]. Although TRIM29 is a member of TRIM family proteins, TRIM29 lacks RING‐finger domain. It has been recently shown that TRIM29 has a remnant E3 ligase activity mediated by its B‐box domains [18]. TRIM29 is highly expressed in many tumor types such as pancreatic, esophageal, bladder, lung, breast, head and neck, and colorectal cancers [19, 20]. It has been reported that TRIM29 knockdown in SiHa, BxPC3, and Panc1 cell lines results in radiosensitivity [21, 22]. In addition, TRIM29 can be phosphorylated by MAPKAP kinase 2 in an ATM‐dependent fashion contributing to radioresistant phenotypes [22]. TRIM29 also interacts with DNA‐PKcs, BRCA1‐associated surveillance complex (BASC), Tat‐interactive protein 60 (TIP60), cohesion, and histone proteins. TRIM29 might function as a scaffold protein for DNA repair protein accumulation at DNA DSB sites resulting in the efficiently active DNA damage response (DDR) [23]. Although previous studies of TRIM29 indicate that it is associated with DNA repair [23, 24], little is known about the roles of TRIM29 in DDR and DNA repair induced by exogenous DNA‐damaging compounds.

To analyze the functions of TRIM29, we disrupted *TRIM29* in DT40 cells. Our data showed that *TRIM29* triallelic knockout (*TRIM29* ^−/−/−/+^) cells were sensitive to etoposide, but resistant to camptothecin. The recruitment of 53BP1 to DNA DSBs was also decreased in *TRIM29* ^−/−/−/+^ cells; however, RAD51 localization was increased. We propose that TRIM29 is a choice regulator of DNA DSB repair pathways by promoting NHEJ and suppressing HR in response to DNA DSBs in vertebrate cells.

## Results

### Generation of *TRIM29* ^−/−/−/+^ cells

**Fig. 1:**
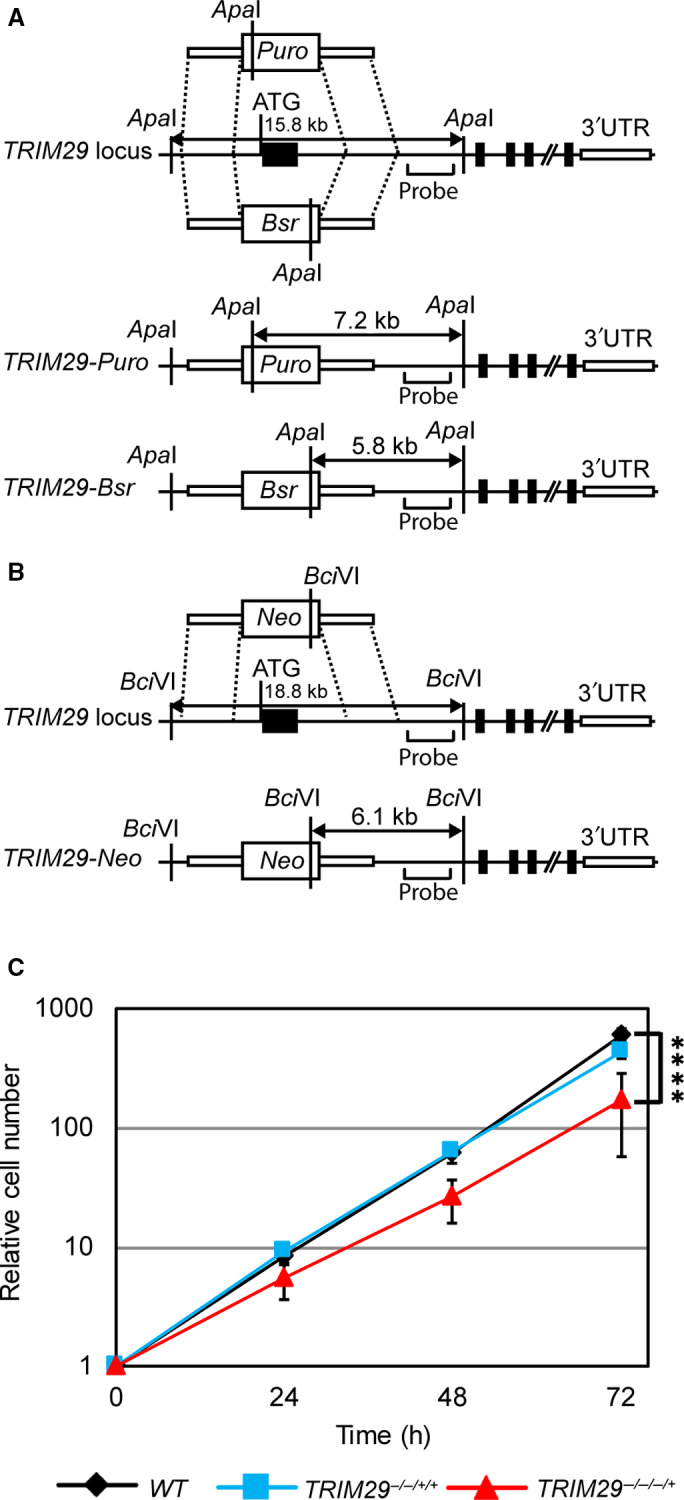
Gene‐targeting constructs of the *TRIM29* locus in DT40 cells and growth kinetics. Schematic representations of a part of the *TRIM29* locus and targeting constructs. The *TRIM29*‐targeting constructs, which contained *Puro*, *Bsr,* or *Neo* selectable markers, were used to disrupt the *TRIM2*9 locus. Black boxes indicate *TRIM29* exons (A, B). Growth kinetics of *WT*, *TRIM29* ^−/−/+/+^, and *TRIM29* ^−/−/−/+^ cells (C). Data are the mean ± SD of three independent experiments (^****^ *P* ≤ 0.0001, relative cell numbers of *WT* cells versus *TRIM29* ^−/−/−/+^ cells, Student’s *t*‐test).

The DT40 cell line is likely tetrasomic at the location of *TRIM29* (chromosome 24), according to copy number variation analysis by the Sequenza package [25]. To generate *TRIM29*‐targeting vectors, a genomic clone containing the *TRIM29* locus was isolated, and a targeting vector was constructed by inserting a selectable gene cassette into the *TRIM29* locus. *TRIM29*‐targeting vectors were expected to replace exon 1 of *TRIM29* (Fig. 1A,B). The targeting vectors were sequentially transfected into wild‐type (*WT)* cells. Disruption of *TRIM29* was validated by Southern blot analysis (Fig. S1A,B) and verified by droplet digital PCR (ddPCR) (Table 1). After multiple transfections, *TRIM29*‐null cells were failed to generate. It might be due to complete knockout of *TRIM29* in DT40 cells is not viable. The rates of gene‐targeting events of *TRIM29* monoallelic knockout (*TRIM29* ^−/+/+/+^), *TRIM29* biallelic knockout (*TRIM29* ^−/−/+/+^), and *TRIM29* ^−/−/−/+^ clones were 2/34 (5.9%), 2/26 (7.7%), and 3/32 (9.38%), respectively. Two clones of *TRIM29* ^−/−/−/+^ were observed (N35 and N46). The results demonstrated that their phenotypes were similar (Table S1 and S2; Figs S3–S5). Therefore, N35 was selected for further observations. *TRIM29* ^−/−/−/+^ cells grew slower than *WT* and *TRIM29* ^−/−/+/+^ cells (Fig. 1C). The doubling times of *WT, TRIM29* ^−/−/+/+^, and *TRIM29* ^−/−/−/+^ cells were 7.7 ± 0.6, 8.2 ± 0.7, and 9.5 ± 1.2 h, respectively.

**Table 1:** Quantification of the *TRIM29* copy number by ddPCR.

Genotype	*TRIM29* (copies·µL^−1^)	Reference gene^a^ (copies µL^−1^)	*TRIM29* copy number (copies)
*WT*	243	135	3.60
*TRIM29* ^−/+/+/+^	194	139	2.79
*TRIM29* ^−/−/+/+^	138	137	2.01
*TRIM29* ^−/−/−/+^ *#N35*	107	236	0.91

^a^ *RNF43^+/+^*. The *TRIM29* copy number was calculated by dividing the concentration of target molecules by that of the reference molecules and multiplying by 2 (the copy number of the *RNF43* gene in DT40 genome).

The cell cycle distribution in asynchronous populations of *WT* and *TRIM29* ^−/−/−/+^ cells was examined by analyzing DNA contents. Under normal conditions, the differences of cell cycle phase distribution between *WT* and *TRIM29^−/−/−/+^* cells were not statistically significant (Table 2 and Fig. S2). Moreover, mitotic indices observed in asynchronous populations of *WT* and *TRIM29* ^−/−/−/+^ cells were not significantly different under normal conditions (Table 3).

**Table 2:** Cell cycle distribution of *WT* and *TRIM29* ^−/−/−/+^ cells under normal conditions (mean ± SD).

Genotype	Sub‐G1 (%)	G1 (%)	S (%)	G2/M (%)
*WT*	1.41 ± 0.20	42.10 ± 4.87	31.58 ± 1.84	24.09 ± 6.92
*TRIM29* ^−/−/−/+^	1.21 ± 0.25^ns^	37.97 ± 1.50^ns^	31.34 ± 1.90^ns^	28.34 ± 3.54^ns^

^ns^ *P* > 0.05, percent of cell cycle phases of *WT* cells versus *TRIM29* ^−/−/−/+^ cells, Student’s *t*‐test.

**Table 3:** Mitotic index of *WT* and *TRIM29* ^−/−/−/+^ cells under normal conditions (mean ± SD).

Genotype	Mitotic index (%)
*WT*	3.54 ± 0.26
*TRIM29* ^−/−/−/+^	3.85 ± 0.60^ns^

^ns^ *P* > 0.05, mitotic index of *WT* cells versus *TRIM29* ^−/−/−/+^ cells, Student’s *t*‐test.

### *TRIM29* ^−/−/−/+^ cells are sensitive to etoposide, but resistant to camptothecin

**Fig. 2:**
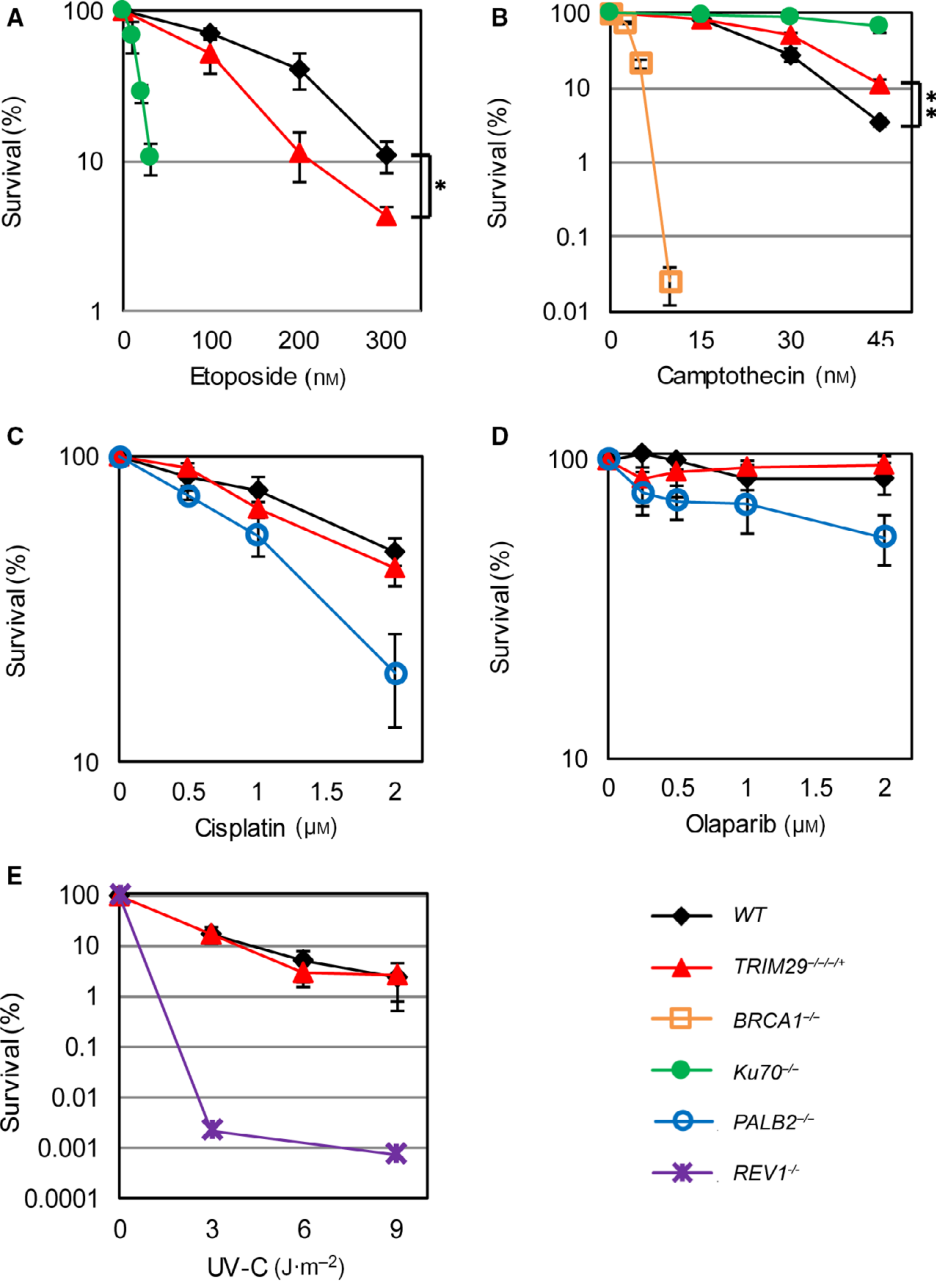
Clonogenic survival assays after genotoxic treatments. Clonogenic survival assays of *WT*, *TRIM29* ^−/−/−/+^, *Ku70* ^−/−^, *PALB2* ^−/−^, *BRCA1* ^−/−^, and *REV1* ^−/−^ cells against etoposide (A), camptothecin (B), cisplatin (C), olaparib (D), and UV‐C (E) treatments. Data are the mean ± SD of three independent experiments (^*^ *P* ≤ 0.05; ^**^ *P* ≤ 0.01, percent survival of *WT* cells versus *TRIM29* ^−/−/−/+^ cells, Student’s *t*‐test).

To investigate the functions of TRIM29 in DNA DSB repair in response to various DNA‐damaging agents, clonogenic survival assays were performed. *TRIM29* ^−/−/−/+^ cells displayed an increased sensitivity to etoposide compared with *WT* cells (Fig. 2A). Etoposide, a TOP2 inhibitor, stabilizes the cleavable TOP2‐DNA complex, thereby inducing DNA DSBs [26]. DNA lesions induced by etoposide are usaully repaired by NHEJ pathway. Surprisingly, *TRIM29* ^−/−/−/+^ cells showed resistance against camptothecin compared with *WT* cells that were similar to NHEJ‐defective cells, such as *Ku70* knockout (*Ku70^−/−^*) cells (Fig. 2B). However, *TRIM29* ^−/−/−/+^ cells were not sensitive to the other DNA‐damaging agents, including cis‐diamminedichloroplatinum (cisplatin), olaparib, or ultraviolet c (UV‐C) (Fig. 2C‐E). These results suggest that TRIM29 is involved in the NHEJ pathway to repair DNA DSBs induced by etoposide.

### *TRIM29* ^−/−/−/+^ cells are defective for DNA DSB repair

**Fig. 3:**
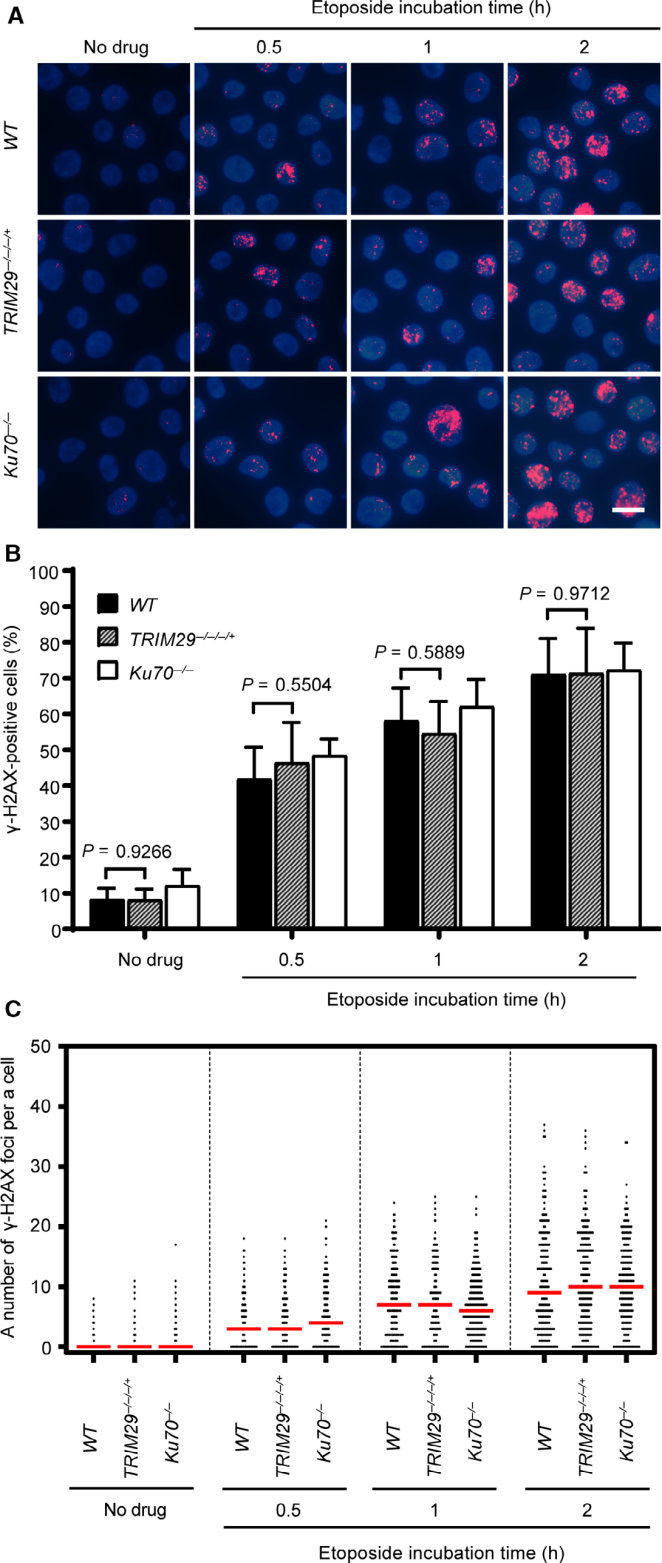
ɣ‐H2AX foci formation induced by 1 µm etoposide treatment. 1 µm etoposide was used to induce ɣ‐H2AX foci formation of each indicated genotype. The formation of ɣ‐H2AX foci was monitored at the indicated time points, shown by representative images (A) and quantification of ɣ‐H2AX‐positive cells, which contain more than four ɣ‐H2AX foci (B), and a number of the ɣ‐H2AX foci per nucleus (C). Median values are indicated in red. Data are the mean ± SD of three independent experiments. The significance in difference between two groups was tested by Student’s *t*‐test. A scale bar, 10 µm. (Figures 3 and 4 represent a single, continuous experiment. The 2‐h time point of Fig. 3 is the same as 0‐h time point in Fig. 4. Some images from certain time points are represented in both figures.)

**Fig. 4:**
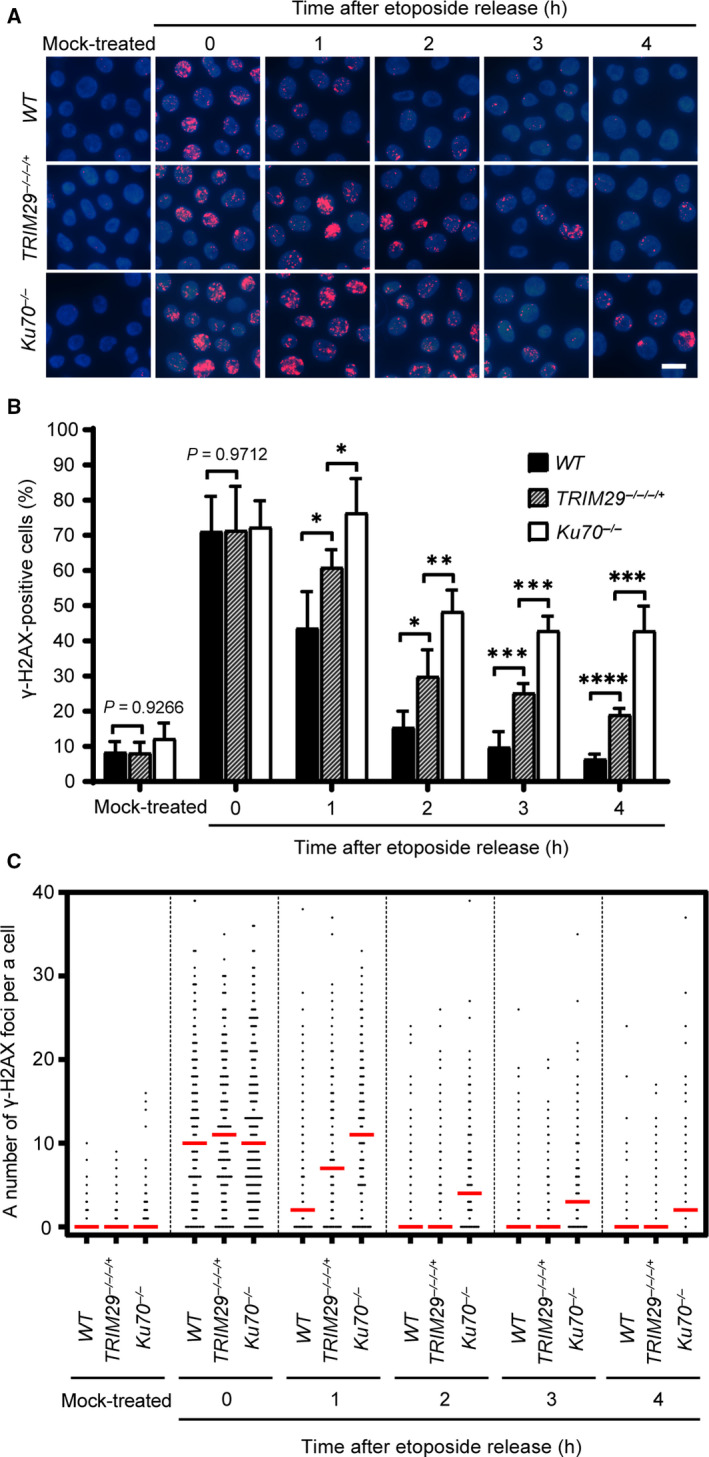
DSB repair kinetics of DNA DSBs induced by etoposide as measured by the dissolution of ɣ‐H2AX foci. ɣ‐H2AX foci formation induced by 1 µm etoposide treatment for 2 h of the indicated genotypes. ɣ‐H2AX foci formation was investigated after the treatment at the indicated time points, shown by representative images (A) and quantification of ɣ‐H2AX‐positive cells, which contain more than four ɣ‐H2AX foci (B), and a number of the ɣ‐H2AX foci per nucleus (C). Median values are indicated in red. Data are the mean ± SD of three independent experiments (^*^ *P* ≤ 0.05; ^**^ *P* ≤ 0.01; ^***^ *P* ≤ 0.001; ^****^ *P* ≤ 0.0001, Student’s *t*‐test). A scale bar, 10 µm. (Figures 3 and 4 represent a single, continuous experiment. The 2 h time point of Fig. 3 is the same as 0 h time point in Fig. 4. Some images from certain time points are represented in both figures.)

**Fig. 5:**
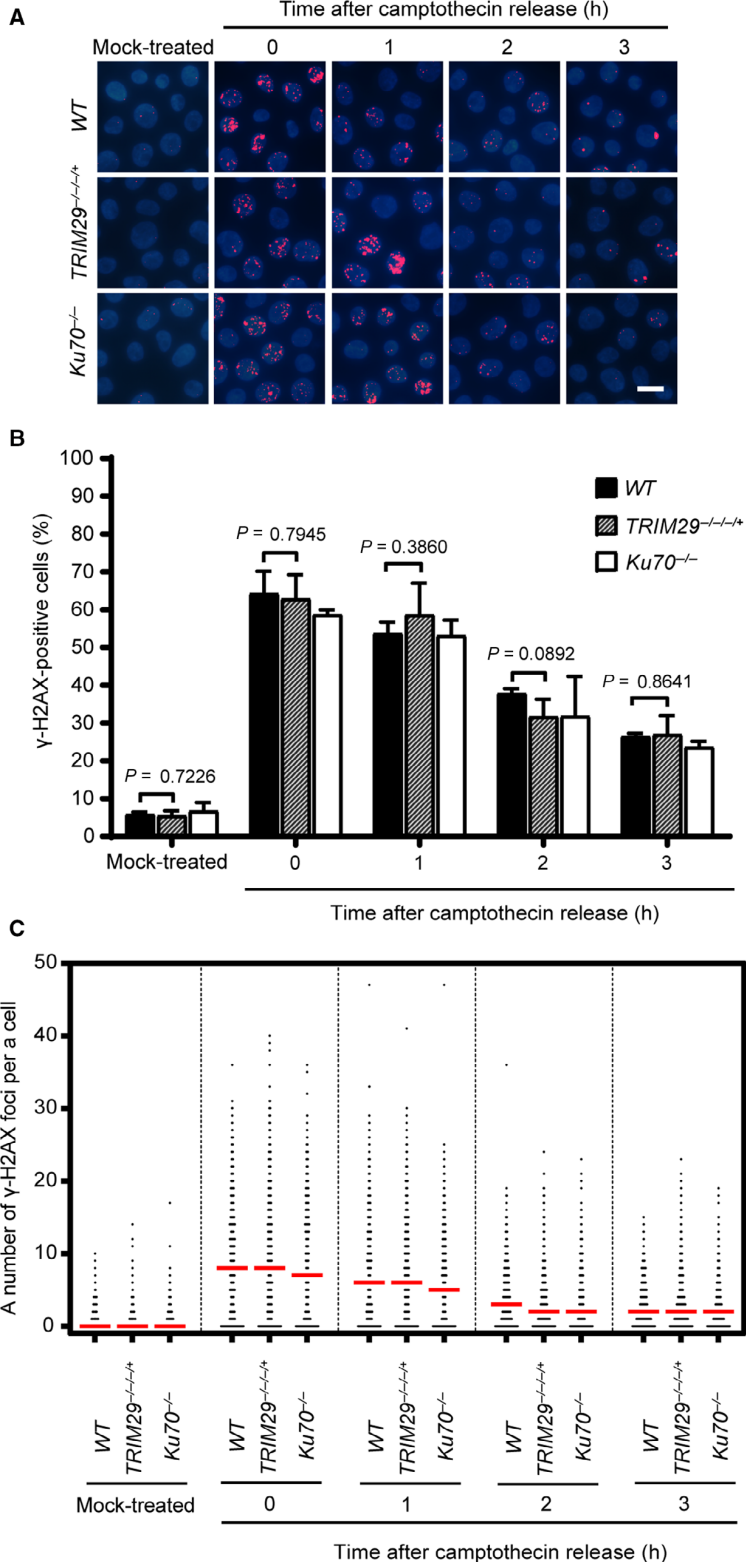
DSB repair kinetics of DNA DSBs induced by camptothecin as measured by the dissolution of ɣ‐H2AX foci. ɣ‐H2AX foci formation induced by 45 nm camptothecin treatment for 3 h of the indicated genotypes. ɣ‐H2AX foci formation was investigated after the treatments at the indicated time points, shown by representative images (A) and quantification of ɣ‐H2AX‐positive cells, which contain more than four ɣ‐H2AX foci (B), and a number of the ɣ‐H2AX foci per nucleus (C). Median values are indicated in red. Data are the mean ± SD of three independent experiments. The significance in difference between two groups was tested by Student’s *t*‐test. A scale bar, 10 µm.

To investigate the DNA DSB repair efficiency in *TRIM29* ^−/−/−/+^ cells, phosphorylated H2A histone family member X at Ser 139 (ɣ‐H2AX) foci formation assays was conducted to measure the DNA DSB repair efficiency induced by etoposide in *WT, TRIM29* ^−/−/−/+^, and *Ku70^−/−^* cells. Cells containing more than four ɣ‐H2AX foci were classified as positive. H2AXs near DNA DSBs are phosphorylated on Ser 139 after formation of DNA DSBs. Therefore, phosphorylation of H2AX on Ser 139 was used as a DNA DSB marker [27]. After pulse treatment with etoposide, ɣ‐H2AX foci formed in a similar fashion in all cell lines (Fig. 3). However, as shown in Fig. 4A–C, the percentages of ɣ‐H2AX‐positive cells and median numbers of ɣ‐H2AX foci per cell of *WT*, *TRIM29* ^−/−/−/+^, and *Ku70^−/−^* cells were decreased in a different time‐dependent manner after pulse‐treated with 1 µm etoposide for 2 h. The delayed in the dissolution of ɣ‐H2AX foci in *TRIM29* ^−/−/−/+^ and *Ku70^−/−^* cells indicating that DNA DSB repair efficiency in response to etoposide treatment of *TRIM29* ^−/−/−/+^ cells was lower than in *WT* cells. In contrast to etoposide, the DNA DSB repair kinetics induced by camptothecin of *WT*, *TRIM29* ^−/−/−/+^, and *Ku70^−/−^* cells were not statistically different (Fig. 5). Taken together, the results suggest that NHEJ‐mediated DSB repair induced by etoposide is defective in *TRIM29* ^−/−/−/+^ cells.

### 53BP1 localization at DSBs is defective in *TRIM29* ^−/−/−/+^ cells

**Fig. 6:**
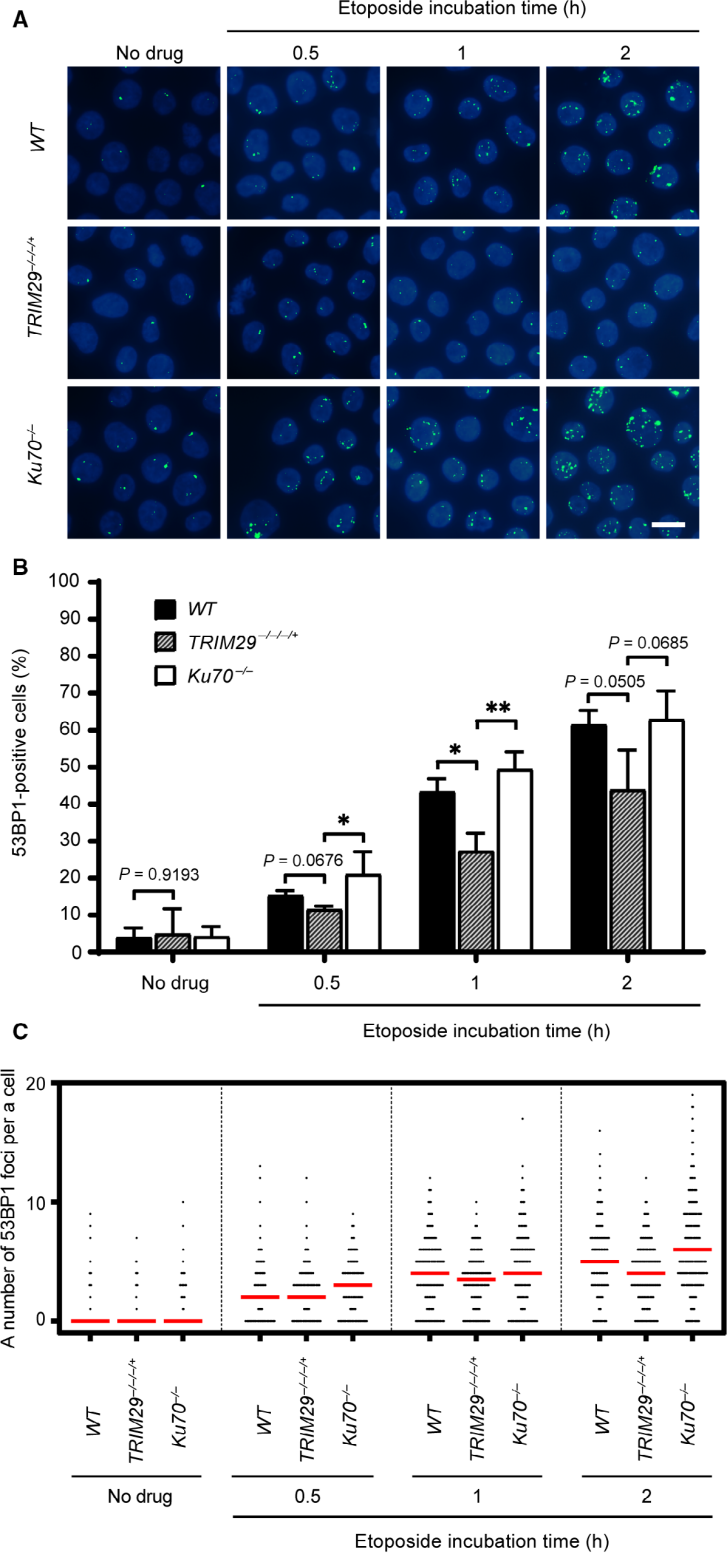
Foci formation of 53BP1 in response to etoposide. 53BP1 foci formation of each genotype induced by 1 µm etoposide was investigated at the indicated time points, shown by representative images (A) and quantification of 53BP1‐positive cells, which contain more than four 53BP1 foci (B), and a number of the 53BP1 foci per nucleus (C). Median values are indicated in red. Data are the mean ± SD of three independent experiments (^*^ *P* ≤ 0.05; ^**^ *P* ≤ 0.01, Student’s *t*‐test). A scale bar, 10 µm.

In response to DNA DSBs, DDR is activated to detect, signal, and recruit DNA repair proteins. Ataxia‐telangiectasia mutated (ATM), ATM’s substrates, mediator of DNA damage checkpoint protein 1 (MDC1), and 53BP1 are key factors in DDR [28]. 53BP1 is one of the important pathway choice regulators of DNA DSB repair pathways by promoting NHEJ and suppressing HR [29, 30]. To investigate the DDR to DNA DSBs, 53BP1 and ɣ‐H2AX foci induced by 1 µm etoposide were monitored at the indicated time points. Cells containing more than four 53BP1 foci were classified as positive. The results showed that the percentages of 53BP1‐positive *TRIM29* ^−/−/−/+^ cells were lower than those of 53BP1‐positive *WT* and *Ku70^−/−^* cells after exposure to etoposide, and the median numbers of 53BP1 foci formation after DNA damage were lower than *WT* and *Ku70^−/−^* cells at 1 and 2 h (Fig. 6), although the percentages of ɣ‐H2AX‐positive *TRIM29* ^−/−/−/+^ cells were not statistically different from those of ɣ‐H2AX‐positive *WT* and *Ku70^−/−^* cells (Fig. 3). These results suggest that the localization of 53BP1 at DNA DSB sites is reduced in *TRIM29* ^−/−/−/+^ cells.

### RAD51 localization at DSBs is elevated in *TRIM29* ^−/−/−/+^ cells

**Fig. 7:**
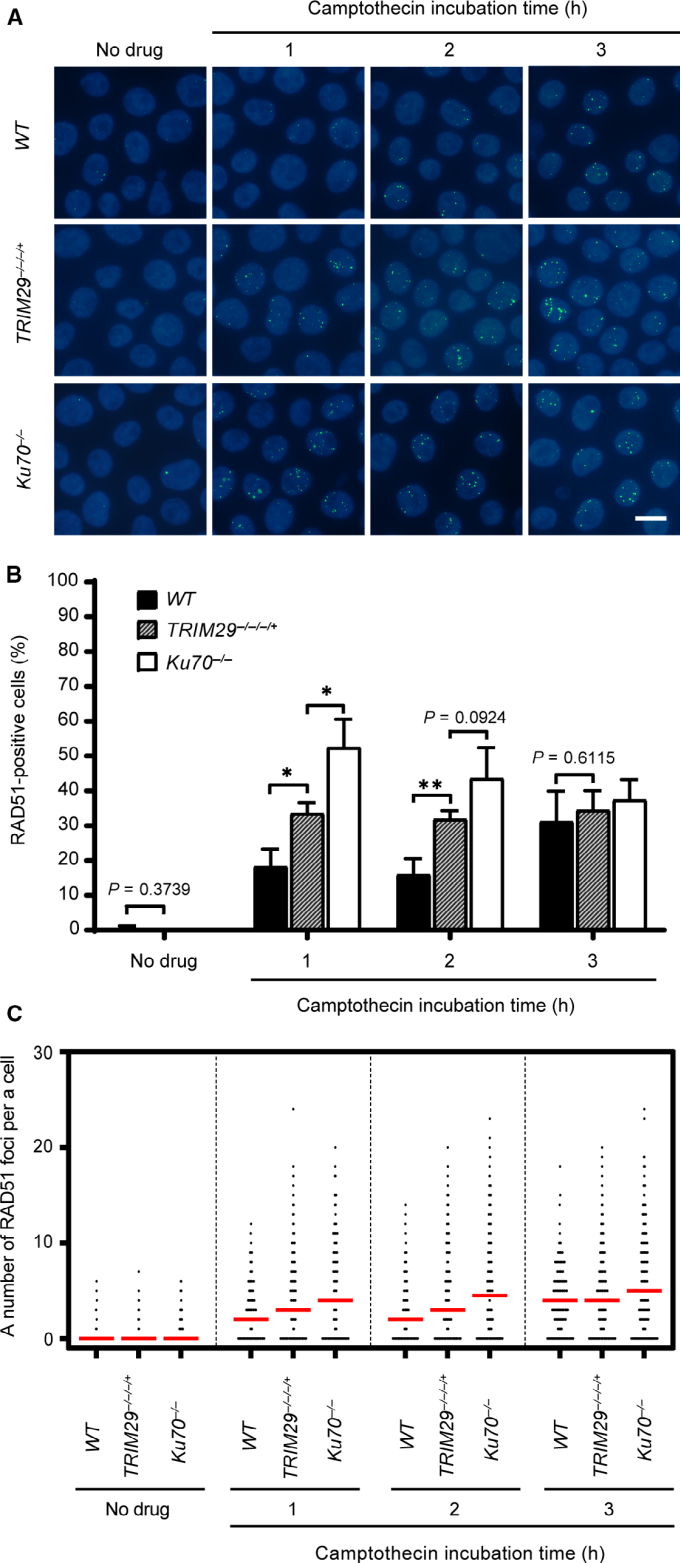
Foci formation of RAD51 in response to camptothecin. 45 nm camptothecin was used to induce RAD51 foci formation of the indicated genotypes. The formation of RAD51 foci was monitored for 3 h during the camptothecin treatment at the indicated time points, shown in by representative images (A) and quantification of RAD51‐positive cells, which contain more than four RAD51 foci (B), and a number of the RAD51 foci per nucleus (C). Median values are indicated in red. Data are the mean ± SD of three independent experiments (^*^ *P* ≤ 0.05; ^**^ *P* ≤ 0.01, Student’s *t*‐test). A scale bar, 10 µm. (Figures 7 and 8 represent a single experiment using different antibodies. Some nuclear staining images are shown in both figures for the same genotype.)

**Fig. 8:**
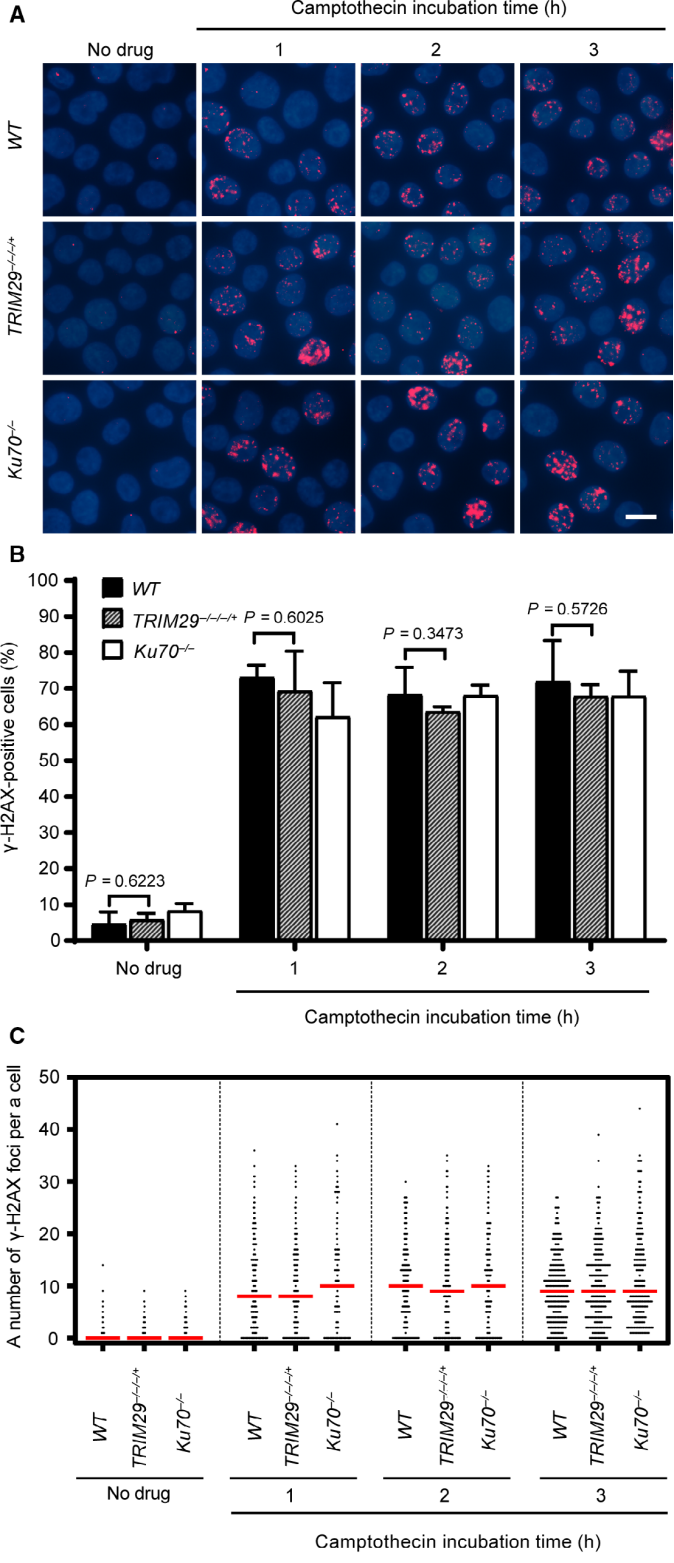
ɣ‐H2AX foci formation induced by 45 nm camptothecin treatment. ɣ‐H2AX foci formation induced by 45 nm camptothecin treatment of the indicated genotypes. The formation of ɣ‐H2AX foci was monitored for 3 h during the camptothecin treatment at the indicated time points, shown by representative images (A) and quantification of ɣ‐H2AX‐positive cells, which contain more than four ɣ‐H2AX foci (B), and a number of the ɣ‐H2AX foci per nucleus (C). Median values are indicated in red. Data are the mean ± SD of three independent experiments. The significance in difference between two groups was tested by Student’s *t*‐test. A scale bar, 10 µm. (Figures 7 and 8 represent a single experiment using different antibodies. Some nuclear staining images are shown in both figures for the same genotype.)

*TRIM29* ^−/−/−/+^ cells were resistant to camptothecin, and 53BP1 failed to efficiently localize to DNA DSBs in *TRIM29* ^−/−/−/+^ cells. To investigate activity of the HR pathway in *TRIM29* ^−/−/−/+^ cells, camptothecin‐induced RAD51 foci were monitored. Cells containing more than four RAD51 foci were classified as positive. As shown in Fig. 7A–C, the percentages of RAD51‐positive *TRIM29* ^−/−/−/+^ were greater than those of RAD51‐positive *WT* cells. The same effect was also observed in *Ku70^−/−^* cells at 1 and 2 h after exposure to camptothecin. We found that the difference in RAD51 recruitment between *WT* and *TRIM29* ^−/−/−/+^ cells was not correlated with DNA damage as the number of ɣ‐H2AX‐positive *TRIM29* ^−/−/−/+^ cells were not significantly different from *WT* and *Ku70^−/−^* after camptothecin treatment (Fig. 8). These results showed that the kinetics of RAD51 foci formation in *TRIM29* ^−/−/−/+^ and *Ku70^−/−^* cells induced by camptothecin were faster than *WT* cells. Taken together, our findings indicate that the activity of the DNA DSB‐induced HR pathway is increased in *TRIM29* ^−/−/−/+^ cells.

## Discussion

We investigated which DNA repair pathway involved TRIM29 in the repair of DNA damage induced by exogenous, genotoxic agents using clonogenic survival assays. In this study, we found that TRIM29 was responsible for the repair of etoposide‐induced DNA DSBs. *TRIM29* ^−/−/−/+^ cells displayed the sensitivity to etoposide, but not cisplatin, olaparib, or UV‐C. In general, TOP2 is associated with relaxation, catenation/decatenation, and winding/unwinding of the DNA double helix to resolve topological entanglement by forming a reversible, cleavable complex with a DNA molecule to generate transient DNA DSBs and then religating DNA ends at the end of this reaction. Etoposide stabilizes the cleavable complex leading to DNA DSBs [26]. Etoposide‐mediated DSBs are predominantly repaired by the NHEJ pathway [31]. Interestingly, *TRIM29* ^−/−/−/+^ cells were resistant to camptothecin that induces an irreversible, cleavable complex of TOP1‐DNA. Generally, the function of TOP1 is to unwind supercoiled DNA molecules associated with DNA replication by inducing DNA single‐strand breaks (SSBs) [32]. When the irreversible TOP1‐DNA complex encounters a replication fork, the unrepaired SSB is converted into a DSB. Thus, camptothecin cytotoxicity is specific to S phase [33, 34]. TOP1‐mediated DNA DSBs generated by camptothecin are preferentially repaired by the HR pathway [35]. Likewise, NHEJ‐defective DT40 cells, such as *Ku70^−/−^*, *DNA‐PKcs* knockout (*DNA*‐*PKcs^−/−/−^*), and *LIG4* knockout (*LIG4^−/−^*) cells, are hypersensitive to etoposide, and NHEJ deficiency confers camptothecin resistance in DT40 cells [31, 36]. Our results indicated that TRIM29 might be involved in NHEJ pathway.

It has been reported that the retention of ɣ‐H2AX foci indicates defects in DNA DSB repair and is correlated with loss of the clonogenic potential [37, 38]. The retention of ɣ‐H2AX foci in *TRIM29* ^−/−/−/+^ cells was observed when *TRIM29* ^−/−/−/+^ cells were treated with etoposide, not camptothecin. It suggested that TRIM29‐deficient cells were defective for etoposide‐induced DNA DSB repair. Using ɣ‐H2AX, 53BP1, and RAD51 foci formation assays, we demonstrated that TRIM29 was involved in 53BP1 localization at DNA DSB sites induced by etoposide. 53BP1 protein functions upstream of NHEJ. It has the ability to promote the NHEJ pathway by inhibiting end resection, a key step in the HR pathway [29, 39]. Furthermore, increased recruitment of RAD51, a key factor of the HR pathway, after exposure of *TRIM29* ^−/−/−/+^ cells to camptothecin was similar to the previous observation of RAD51 foci formation induced in *XRCC4* mutant fibroblasts that were defective in the NHEJ pathway. Bee et al. reported that recruitment of RAD51 at DNA DSB sites induced by IR increased in *XRCC4* mutant fibroblasts compared with *WT* fibroblasts. This compensation of HR in NHEJ‐defective cells is normally observed in higher eukaryotes [8]. Moreover, the analysis of HR‐mediated DNA DSB repair using I‐*Sce*I‐based assays reveals that the frequency of HR repair is increased in *Ku70* ^−/−^, *XRCC4* knockout (*XRCC4^−/−^*), and *DNA‐PKcs* ^−/−^ embryonic stem cell lines [40]. This phenomenon probably proceeds via error‐free RAD51‐mediated gene conversion (GC) rather than mutagenic RAD52‐mediated SSA as recent literature suggested SSA might function as an alternative DSB repair pathway in HR‐deficient cells in higher eukaryotic models [3, 41].

Mechanistically, it has been speculated that 53BP1 and other NHEJ factors that suppress HR by inhibiting DNA end resection at DSB sites need to be removed by BRCA1 [42, 43]. Therefore, HR factors bind to DNA DSBs with less competition in NHEJ‐defective cells. Collectively, our data suggest that TRIM29 is essential for the recruitment of 53BP1 to promote the NHEJ pathway, thereby suppressing the HR pathway. However, the mechanism underlying how TRIM29 facilitates 53BP1 recruitment at DNA DSB sites is unclear. It has been reported that a physical interaction between TRIM29 and RNF8 promotes DNA DSB repair [24], and overexpression of TRIM29 inhibits TIP60 functions by stimulating degradation and changing the localization of TIP60 [44]. In addition, TIP60 suppresses the binding of 53BP1 to histone H4 dimethylated at Lys 20 (H4K20^Me2^) by acetylating of histone H4 on Lys 16 (H4K16^Ac^) [45, 46]. Therefore, TRIM29 may facilitate the recruitment of 53BP1 to DNA DSBs by two possible mechanisms. One mechanism involves TRIM29 assisting RNF8 in the RNF8‐RNF168 pathway to remove JMJD2A and L3MBTL1 that compete with 53BP1 for H4K20^Me2^ [47, 48], allowing 53BP1 to bind to H4K20^Me2^. The other mechanism involves TRIM29 diminishing TIP60‐mediated H4K16 acetylation, leading to an increase in 53BP1 binding to H4K20^Me2^.

## Conclusion

In summary, whether TRIM29 functions through RNF8 or TIP60 in order to facilitate choice of DNA DSB repair pathways remain unknown. Further functional studies on TRIM29 using additional models will help elucidate molecular functions of TRIM29 in DNA DSB repair. According to this study, TRIM29 acts as one of DNA DSB repair pathway choice regulators, promoting 53BP1 recruitment to DNA DSB sites. The better understanding of functions of TRIM29 may facilitate establishment of new cancer treatments. For instance, targeting TRIM29 may sensitize refractory cancers to therapies. Although we did not clearly clarify the association of TRIM29 with DNA DSB repair, this study defines a novel role of TRIM29 in facilitating NHEJ and sheds light on TRIM29 in the field of DNA repair.

## Materials and methods

### Cell lines and cultures

*WT*, *Ku70^−/−^*, *BRCA1* knockout (*BRCA1^−/−^)*, *PALB2* knockout (*PALB2^−/−^)*, and *REV1* knockout (*REV1^−/−^)* DT40 cells were kind gifts from Prof. Shunichi Takeda (Department of Radiation Genetics, Graduate School of Medicine, Kyoto University). DT40 cells were cultured in RPMI 1640 medium (Gibco, Gaithersburg, MD, USA), supplemented with 10% fetal bovine serum (Gibco), 1% chicken serum (Gibco), 2 mm L‐glutamine (Gibco), 100 µm β‐mercaptoethanol (Gibco), and 100 U·mL^−1^ penicillin/streptomycin (Gibco), at 40 °C with 5% CO_2_. All cultures were maintained in the exponential phase of growth [49]. Mutants were generated using *WT* cells.

### Construction of targeting vectors

To construct *TRIM29* (Gene ID:419754)‐targeting vectors, genomic DNA sequences were amplified with two sets of primers, 5′‐CGCGGTGGCGGCCGCTCTAGAAGCTTCCTTGCAGC AGGGAC‐3′ and 5′‐TGATCACATTTAAGTGTCATATCAAGCAACTTCG‐3′, and 5′‐CTTG ATATGACACTTAAATGTGATCATGCAGACAGAGAAAGTGCAGCTTA‐3′ and 5′‐CTGG GTACCGGGCCCCCCCAACACTGGTATGGATAGCAGCAAG‐3′ for left and right arms, respectively. Both amplified DNA fragments (3 and 2 kb for left and right arms, respectively) were inserted into a digested pBlueScript II SK(+) vector using NEBuilder HiFi (New England Biolabs, Ipswich, MA, USA). The *BamH*I restriction site was used to insert selectable marker cassettes, puromycin acetyltransferase (*Puro*), blasticidin S deaminase (*Bsr*), or neomycin phosphotransferase (*Neo*) selectable markers [50]. The 0.8‐kb DNA fragment amplified from genomic DNA using primers: 5′‐TCAATGGCTCTCAGATGCAG‐3′ and 5′‐ACAAAAGGAAGAGGGGA GGA‐3′ was used as a probe for Southern blot analysis to screen gene‐targeting events. *TRIM29*‐targeting vectors, *TRIM29*‐*Puro*, *TRIM29*‐*Bsr* and *TRIM29*‐*Neo*, were linearized with *Kpn*I prior to transfection.

### Transfection and establishment of gene‐disrupted cells

Transfection was conducted as described previously [51]. Briefly, 1 × 10^7^ cells were electroporated with 30 µg linearized *TRIM29*‐targeting vectors using a Genepulser Xcell™ (Bio‐Rad Life Sciences, Hercules, CA, USA). After electroporation, the cells were cultured in 20 mL prewarmed RPMI 1640 medium at 40 °C with 5% CO_2_ for 24 h and then diluted with 60 mL drug‐containing RPMI 1640 medium. The final concentrations of each antibiotic were 0.5 µg·mL^−1^ puromycin dihydrochloride (Toku‐E, Bellingham, WA, USA), 25 µg·mL^−1^ blasticidin S HCl (Toku‐E), and 2 mg·mL^−1^ G418 disulfate (PanReac AppliChem, Darmstadt, Germany). Successful targeting events of drug‐resistant colonies were determined by Southern blot analysis as described previously [52]. *WT* cells were sequentially transfected with *TRIM29*‐*Puro*‐, *TRIM29*‐*Bsr*‐, and *TRIM29*‐*Neo*‐targeting vectors to obtain *TRIM29* ^−/+/+/+^, *TRIM29* ^−/−/+/+^, and *TRIM29* ^−/−/−/+^ cells, respectively (Fig. 1A,B). Targeting events of *TRIM29* ^−/−/−/+^ cells were identified by the appearance of 5.8, 7.1, 7.2, and 15.8 kb bands of *Apa*I‐digested genomic DNA as well as 6.1 and 18.8 kb bands in *Bci*VI‐digested genomic DNA in Southern blot analysis.

### Droplet digital PCR assay

Two sets of primers were used in ddPCR, 5′‐AGTGAGCTTCTGCC TTCTTGTTTG‐3′ and 5′‐AGACTGTTGTAGTAACACTTCAGGG‐3′ for *TRIM29*, and 5′‐GTGTTGACTAAGGGA AGGCTTGAAC‐3′ and 5′‐CCACACAGTCCTTCTTTCATTGTCC‐3′ for *RNF43* to determine the copy number of *TRIM29* in *WT* cells and mutants, according to the manufacturer’s instructions, and quantasoft droplet reader software, version 1.6.6.0320 (Bio‐Rad Life Sciences) was used to analyze the data.

### Cell proliferation assay

For the proliferation assay, cells were seeded in 24‐well plates and cultured for 72 h. The cells were maintained in the exponential phase of growth. The number of cells was counted using an improved Neubauer hemocytometer with trypan blue (Gibco) every 24 h. Three independent experiments were conducted.

### Clonogenic survival assays

Clonogenic survival assays were conducted as described previously [53]. Briefly, 1 × 10^2−5^ cells were seeded in 6‐well plates containing 6 mL of 1.6% W/V methylcellulose‐containing DMEM/F12 medium (Gibco) with various concentrations of DNA‐damaging agents: etoposide (0, 100, 200, and 300 nm) (Sigma‐Aldrich, St. Louis, MO, USA), camptothecin (0, 15, 30, and 45 nm) (Sigma‐Aldrich), cisplatin (0, 0.5, 1, 1.5, and 2 µm) (Sigma‐Aldrich), and olaparib (0, 0.5, 1, 1.5, and 2 µm) (Selleckchem, Houston, TX, USA). For UV‐C sensitivity assay, cells were seeded as described above and exposed to various doses of UV‐C (0, 3, 6, and 9 J·m^−2^) using a HL‐2000 HybridLinker™ hybridization oven and cross‐linker (Thermo Fisher Scientific, Waltham, MA, USA), and then, 6 mL of 1.6% W/V methylcellulose‐containing DMEM/F12 medium was added. Surviving colonies were counted within 10–14 days. The percentage of survival was calculated by normalizing to a number of surviving colonies of untreated cells.

### ɣ‐H2AX, 53BP1, and RAD51 foci formation assays

Briefly, 7 × 10^5^ cells were treated with 1 µm etoposide for 2 h or 45 nm camptothecin for 3 h and then washed with phosphate‐buffered saline (PBS) twice and resuspended in fresh medium. Cells were harvested onto the surface of a glass slide at the indicated time points using a Cytospin4 cytocentrifuge (Thermo Fisher Scientific), fixed with 4% paraformaldehyde for 10 min, permeabilized with 0.5% Triton X‐100 for 10 min, and blocked in Odyssey^®^ blocking buffer (LI‐COR Biosciences, Lincoln, NE, USA) at room temperature for 1 h. The cells were then incubated with mouse monoclonal anti‐ɣ‐H2AX, Ser 139 antibody (1:500; CST, cat. #80312, Danvers, MA, USA), and rabbit polyclonal anti‐53BP1 antibody (1:500; Novus Biologicals, cat. #NB100‐904, Centennial, CO, USA) for the etoposide‐induced foci formation assay, or rabbit monoclonal anti‐ɣ‐H2AX, Ser 139 antibody (1:1000; CST, cat. #9718) and mouse monoclonal anti‐RAD51 antibody (1:200; Santa Cruz, cat. #sc‐398587, Dallas, TX, USA) for the camptothecin‐induced foci formation assay at room temperature for 1 h. After intensive washing, the cells were incubated with goat anti‐mouse IgG conjugated with Dylight 594 (1:500; Thermo Fisher Scientific, cat. #35510) and goat anti‐rabbit IgG conjugated with Dylight 488 (1:500; Thermo Fisher Scientific, cat. #35552), and donkey anti‐rabbit IgG conjugated with Alexa Fluor 594 (1:500; Thermo Fisher Scientific, cat. #A‐21207) and donkey anti‐mouse IgG conjugated with Alexa Fluor 488 (1:500; Thermo Fisher Scientific, cat. #A‐21202) for etoposide‐ and camptothecin‐induced foci formation assays, respectively, at room temperature for 1 h. Cells were counterstained with Hoechst 33258 (1:2000; Thermo Fisher Scientific), and foci of ɣ‐H2AX, 53BP1, and RAD51 were observed under a fluorescence microscope from Nikon (Eclipse Ci Series, Tokyo, Japan). All samples were visualized using the same exposure time and intensity. At least 100 cells of each treatment were analyzed using photoshop cc2018 version 19.1.0 (Adobe, San Jose, CA, USA). For 53BP1 foci formation assay, 53BP1 nuclear bodies were not counted [54].

### Determination of the cell cycle distribution by flow cytometry and mitotic index

To analyze the cell cycle phase distribution, cells were washed with PBS and fixed with 70% ice‐cold ethanol at −20 °C for at least 2 h. The cells were then stained with propidium iodide from a Muse^®^ Cell cycle Assay Kit (Merck Millipore, Burlington, MA, USA), according to the manufacturer’s protocol. The cell cycle distribution was analyzed by flow cytometry using a Navios flow cytometer and kaluza analysis 2.1 software (Beckman Coulter, Inc., Brea, CA, USA). The mitotic index was calculated by a number of cells undergoing mitosis divided by a total number of cells, which were manually counted from fluorescence images at least 1000 cells.

### Statistical analysis

Comparisons between groups were made by Student’s *t*‐test using graphpad prism 7 (Software, Inc., La Jolla, CA, USA). Results are displayed as the mean ± standard deviation (SD) or median. Statistical significance was accepted at *P < *0.05.

## Conflict of interest

The authors declare no conflict of interest.

## Author contributions

RW wrote the manuscript and prepared all figures. RW, TL, WS, and SC performed experiments. All data were analyzed by RW and DD. RW, TL, and DD designed the study. DD and TS provided supervision and obtained funding. All authors read and approved the final manuscript.

## Supporting information

Fig. S1. Southern blot analysis for *TRIM29^‐/‐/‐/+^* screening. Southern blot analyses of *Bci*VI‐digested genomic DNA (A) and *Apa*I‐digested genomic DNA (B). *TRIM29^‐/‐/‐/+^* clones were identified by the appearance of 6.1 and 18.8 kb bands in Southern blots of *Bci*VI‐digested genomic DNA and 5.8, 7.1, 7.2 and 15.8 kb bands in Southern blots of *Apa*I‐digested genomic DNA. N13, N26 and N35 were candidates for *TRIM29^‐/‐/‐/+^* cells. N13, N26 and N35 were checked for the *TRIM29* copy number by ddPCR. The results of ddPCR confirmed that N35 was *TRIM29^‐/‐/‐/+^*.
**Fig. S2.** Cell cycle analysis. Representative FACS analysis of *WT* (A) and *TRIM29^‐/‐/‐/+^* (B).
**Fig. S3.** Growth kinetics of *TRIM29^‐/‐/‐/+ ^#N46*. The growth kinetic of *TRIM29^‐/‐/‐/+^* *#N46* compared with *WT*, *TRIM29^‐/‐/+/+^* and *TRIM29^‐/‐/‐/+^ #N35*. Data are the mean ± S.D. of three independent experiments (^****^ *P* ≤ 0.0001, relative cell numbers of *WT* cells versus *TRIM29* ^‐/‐/‐/+^ *#N35* cells, Student’s *t*‐test).
**Fig. S4.** Clonogenic survival assays after etoposide treatments. Clonogenic survival assays of *WT*, *TRIM29* ^‐/‐/‐/+^ *#N35*, *TRIM29* ^‐/‐/‐/+^ *#N46* and *Ku70* ^‐/‐^ cells against etoposide treatments. Data are the mean ± S.D. of three independent experiments (^***^ *P* ≤ 0.001, Student’s *t*‐test).
Fig. S5. Foci formation of 53BP1 in response to etoposide. 53BP1 foci formation of *WT*, *TRIM29* ^‐/‐/‐/+^ *#N35*, *TRIM29* ^‐/‐/‐/+^ *#N46* and *Ku70* ^‐/‐^ cells induced by 1 µM etoposide was investigated at the indicated time points, shown by representative images (A) and quantification of 53BP1‐positive cells, which contain more than four 53BP1 foci (B), and a number of the 53BP1 foci per nucleus (C). Median values are indicated in red. Data are the mean ± S.D. of three independent experiments (^*^ *P* ≤ 0.05; ^**^ *P* ≤ 0.01; ^***^ *P* ≤ 0.001, Student’s *t*‐test). A scale bar, 10 µm.
**Table S1.** Quantification of the *TRIM29* copy number in 2 clones of *TRIM29* ^‐/‐/‐/+^ by ddPCR.
**Table S2.** Doubling time of *WT* and mutants.

## Data Availability

Raw data are available from the corresponding author upon reasonable request.
