# Supplementary material for: TRIM29 is required for efficient recruitment of 53BP1 in response to DNA double‐strand breaks in vertebrate cells
Source: FEBS Open Bio. 2020 Aug 31;10(10):2055–71. doi: 10.1002/2211-5463.12954 (PMC7530400; doi:10.1002/2211-5463.12954)
Supplement: Supplementary file 1 — Fig. S1. Southern blot analysis for TRIM29‐/‐/‐/+ screening. Southern blot analyses of BciVI‐digested genomic DNA (A) and ApaI‐digested genomic DNA (B). TRIM29‐/‐/‐/+ clones were identified by the appearance of 6.1 and 18.8 kb bands in Southern blots of BciVI‐digested genomic DNA and 5.8, 7.1, 7.2 and 15.8 kb bands in Southern blots of ApaI‐digested genomic DNA. N13, N26 and N35 were candidates for TRIM29‐/‐/‐/+ cells. N13, N26 and N35 were checked for the TRIM29 copy number by ddPCR. The results of ddPCR confirmed that N35 was TRIM29‐/‐/‐/+. Fig. S2. Cell cycle analysis. Representative FACS analysis of WT (A) and TRIM29‐/‐/‐/+ (B). Fig. S3. Growth kinetics of TRIM29‐/‐/‐/+ #N46. The growth kinetic of TRIM29‐/‐/‐/+ #N46 compared with WT, TRIM29‐/‐/+/+ and TRIM29‐/‐/‐/+ #N35. Data are the mean ± S.D. of three independent experiments (**** P ≤ 0.0001, relative cell numbers of WT cells versus TRIM29 ‐/‐/‐/+ #N35 cells, Student’s t‐test). Fig. S4. Clonogenic survival assays after etoposide treatments. Clonogenic survival assays of WT, TRIM29 ‐/‐/‐/+ #N35, TRIM29 ‐/‐/‐/+ #N46 and Ku70 ‐/‐ cells against etoposide treatments. Data are the mean ± S.D. of three independent experiments (*** P ≤ 0.001, Student’s t‐test). Fig. S5. Foci formation of 53BP1 in response to etoposide. 53BP1 foci formation of WT, TRIM29 ‐/‐/‐/+ #N35, TRIM29 ‐/‐/‐/+ #N46 and Ku70 ‐/‐ cells induced by 1 µM etoposide was investigated at the indicated time points, shown by representative images (A) and quantification of 53BP1‐positive cells, which contain more than four 53BP1 foci (B), and a number of the 53BP1 foci per nucleus (C). Median values are indicated in red. Data are the mean ± S.D. of three independent experiments (* P ≤ 0.05; ** P ≤ 0.01; *** P ≤ 0.001, Student’s t‐test). A scale bar, 10 µm. Table S1. Quantification of the TRIM29 copy number in 2 clones of TRIM29 ‐/‐/‐/+ by ddPCR. Table S2. Doubling time of WT and mutants. [file FEB4-10-2055-s001.docx]

**Table S1** Quantification of the *TRIM29* copy number in 2 clones of *TRIM29*^-/-/-/+^ by ddPCR

| **Genotype** | ***TRIM29*** | **Reference gene^£^** | ***TRIM29* copy number** |
| --- | --- | --- | --- |
|  | **(copies µl^-1^)** | **(copies µl^-1^)** | **(copies)** |
| *TRIM29*^-/-/-/+^ *#N35* | 44.7 | 101 | 0.88 |
| *TRIM29*^-/-/-/+^ *#N46* | 13.7 | 32.5 | 0.84 |
| ^£^*RNF43^+/+^.* The *TRIM29* copy number was calculated by dividing the concentration of target molecules by that of the reference molecules and multiplying by 2 (the copy number of the *RNF43* gene in DT40 genome). | | | |

**Table S2** Doubling time of *WT* and mutants

| **Genotype** | **Doubling time (h)** |
| --- | --- |
| *WT* | 7.7 ± 0.6 |
| *TRIM29*^-/-/+/+^ | 8.2 ± 0.7 |
| *TRIM29*^-/-/-/+^ *#N35* | 9.5 ± 1.2 |
| *TRIM29*^-/-/-/+^ *#N46* | 10.2 ± 1.4 |


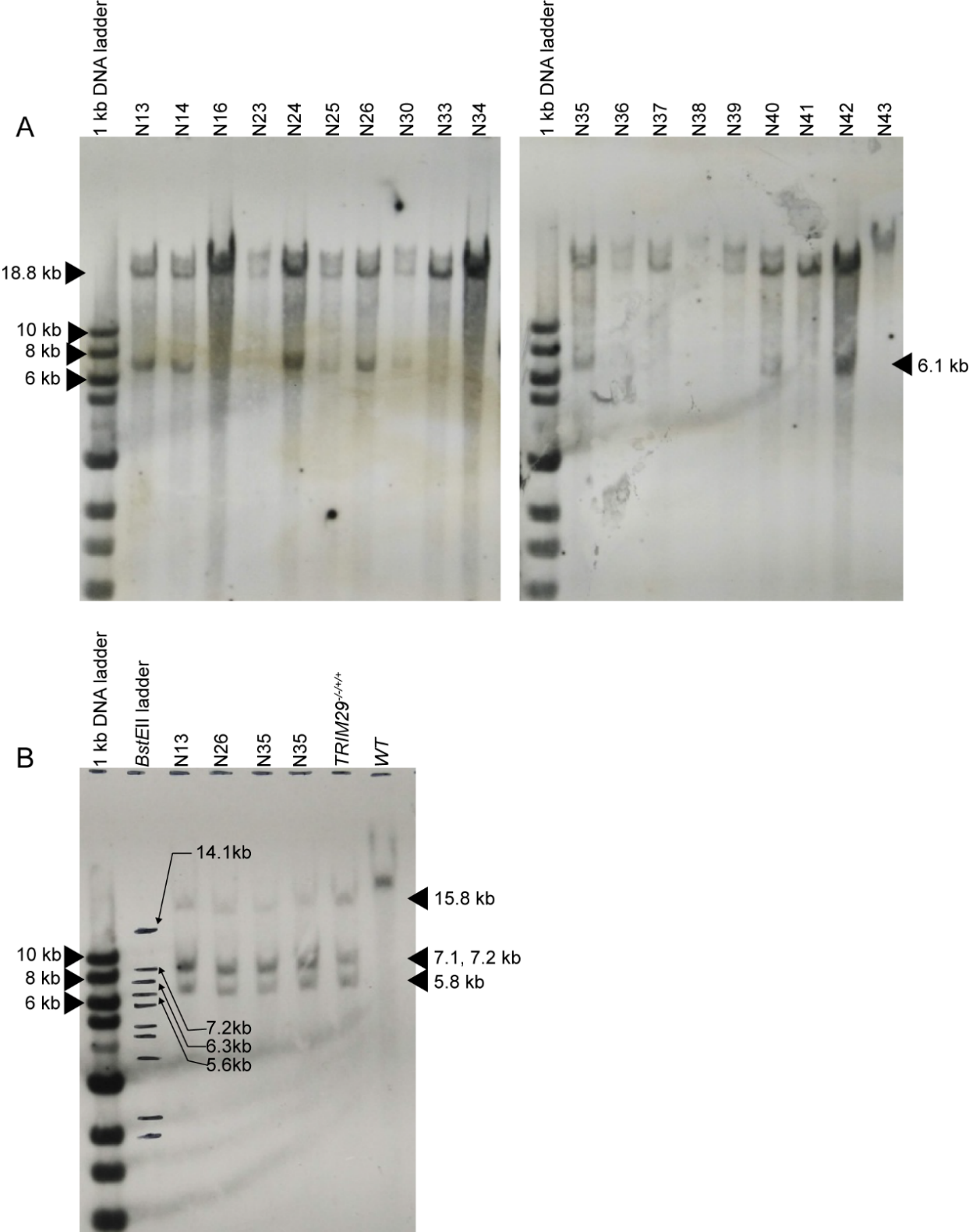


**Fig. S1** **Southern blot analysis for *TRIM29^-/-/-/+^* screening.** Southern blot analyses of *Bci*VI-digested genomic DNA (A) and *Apa*I-digested genomic DNA (B). *TRIM29^-/-/-/+^* clones were identified by the appearance of 6.1 and 18.8 kb bands in Southern blots of *Bci*VI-digested genomic DNA and 5.8, 7.1, 7.2 and 15.8 kb bands in Southern blots of *Apa*I-digested genomic DNA. N13, N26 and N35 were candidates for *TRIM29^-/-/-/+^* cells. N13, N26 and N35 were checked for the *TRIM29* copy number by ddPCR. The results of ddPCR confirmed that N35 was *TRIM29^-/-/-/+^.*


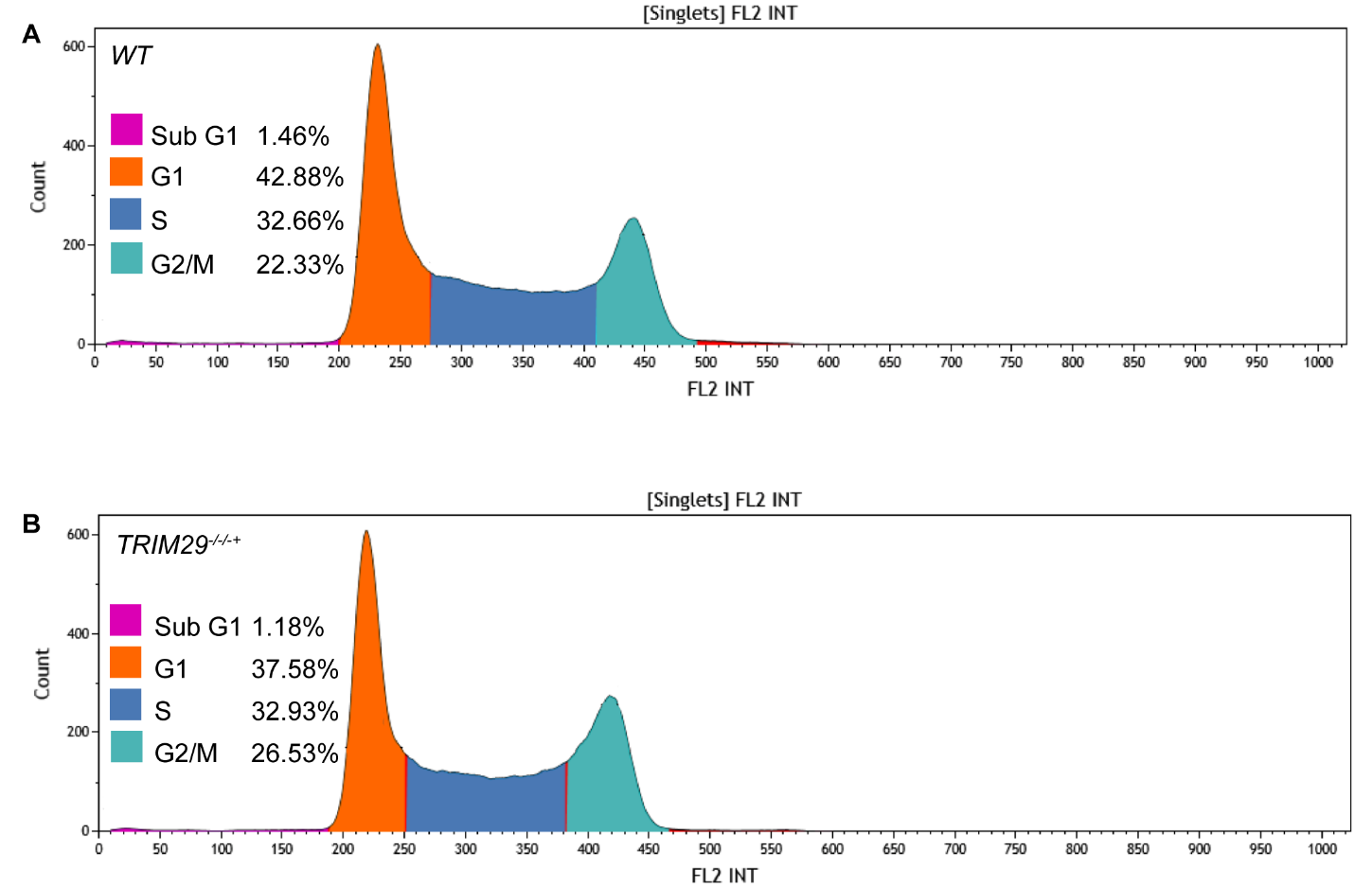


**Fig. S2 Cell cycle analysis.** Representative FACS analysis of *WT* (A) and *TRIM29^-/-/-/+^* (B).


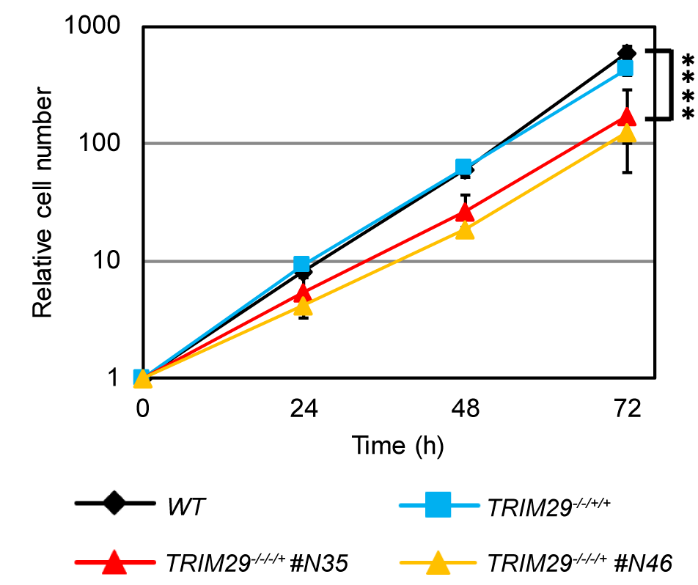


**Fig. S3 Growth kinetics of *TRIM29^-/-/-/+^ #N46*.** The growth kinetic of *TRIM29^-/-/-/+^* *#N46* compared with *WT*, *TRIM29^-/-/+/+^* and *TRIM29^-/-/-/+^ #N35*. Data are the mean ± S.D. of three independent experiments (^****^*P* ≤ 0.0001, relative cell numbers of *WT* cells versus *TRIM29*^-/-/-/+^ *#N35* cells, Student’s *t*-test).


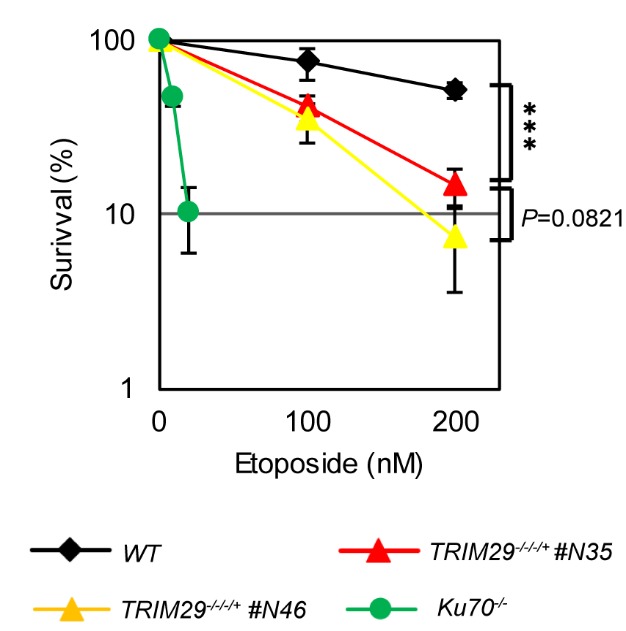


**Fig. S4 Clonogenic survival assays after etoposide treatments**. Clonogenic survival assays of *WT*, *TRIM29*^-/-/-/+^ *#N35*, *TRIM29*^-/-/-/+^ *#N46* and *Ku70*^-/-^ cells against etoposide treatments. Data are the mean ± S.D. of three independent experiments (^***^*P* ≤ 0.001, Student’s *t*-test).


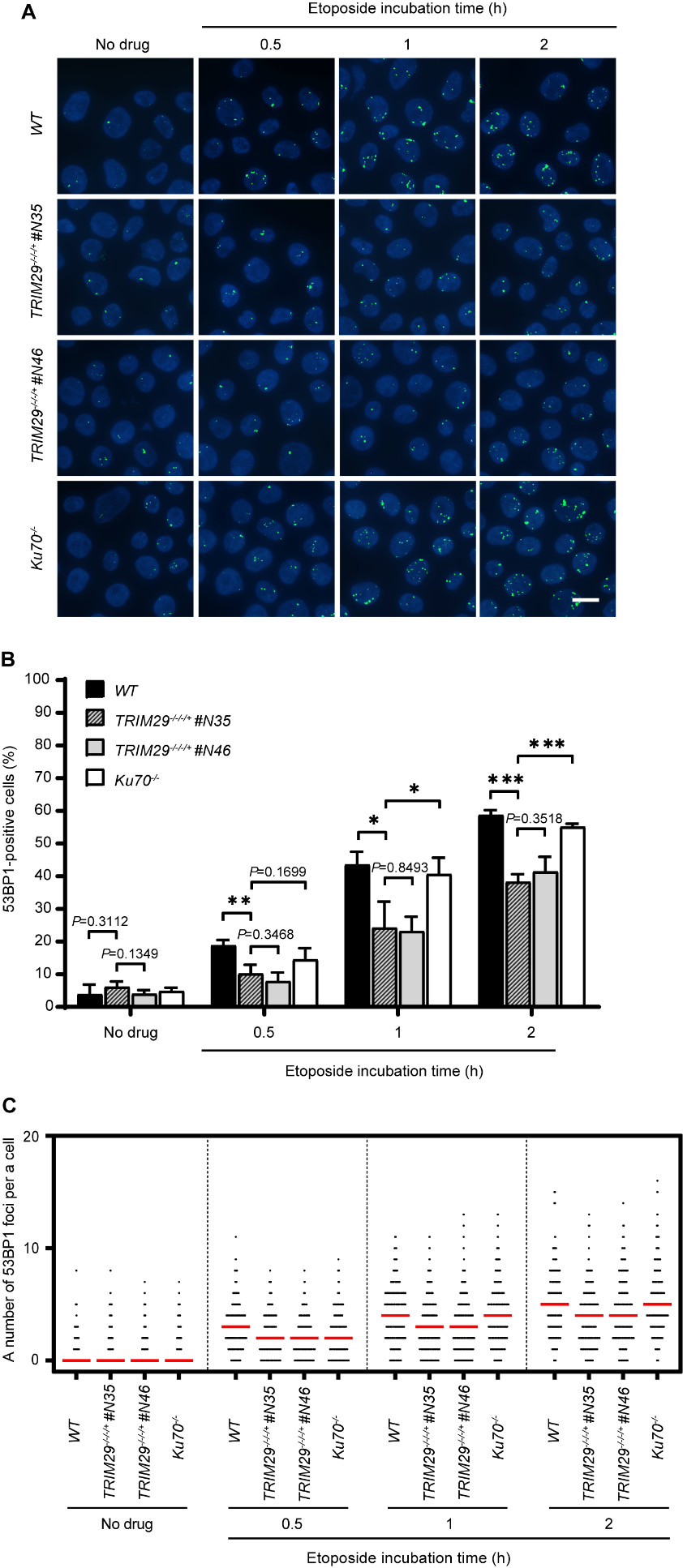


**Fig. S5 Foci formation of 53BP1 in response to etoposide.** 53BP1 foci formation of *WT*, *TRIM29*^-/-/-/+^ *#N35*, *TRIM29*^-/-/-/+^ *#N46* and *Ku70*^-/-^ cells induced by 1 µM etoposide was investigated at the indicated time points, shown by representative images (A) and quantification of 53BP1-positive cells, which contain more than four 53BP1 foci (B), and a number of the 53BP1 foci per nucleus (C). Median values are indicated in red. Data are the mean ± S.D. of three independent experiments (^*^*P* ≤ 0.05; ^**^*P* ≤ 0.01; ^***^*P* ≤ 0.001, Student’s *t*-test). A scale bar, 10 µm.
